# Author Correction: Regulation of local GTP availability controls RAC1 activity and cell invasion

**DOI:** 10.1038/s41467-021-26665-2

**Published:** 2021-11-05

**Authors:** Anna Bianchi-Smiraglia, David W. Wolff, Daniel J. Marston, Zhiyong Deng, Zhannan Han, Sudha Moparthy, Rebecca M. Wombacher, Ashley L. Mussell, Shichen Shen, Jialin Chen, Dong-Hyun Yun, Anderson O’Brien Cox, Cristina M. Furdui, Edward Hurley, Maria Laura Feltri, Jun Qu, Thomas Hollis, Jules Berlin Nde Kengne, Bernard Fongang, Rui J. Sousa, Mikhail E. Kandel, Eugene S. Kandel, Klaus M. Hahn, Mikhail A. Nikiforov

**Affiliations:** 1 Department of Cell Stress Biology, Roswell Park Comprehensive Cancer Center, Buffalo, NY USA; 2 Department of Cancer Biology, Wake Forest University Baptist Medical Center, Winston-Salem, NC USA; 3 Department of Pharmacology and Lineberger Cancer Center, University of North Carolina at Chapel Hill, Chapel Hill, NC USA; 4 New York State Center of Excellence Bioinformatics and Life Sciences, State University of New York at Buffalo, Buffalo, NY USA; 5 Department of Biochemistry and Neurology, Hunter James Kelly Research Institute, Jacobs School of Medicine and Biomedical Sciences, State University of New York at Buffalo, Buffalo, NY USA; 6 Department of Biochemistry, Center for Structural Biology, Wake Forest School of Medicine, Winston-Salem, NC USA; 7 Department of Physics, University of Houston, Houston, TX USA; 8 Glenn Biggs Institute for Alzheimer’s and Neurodegenerative Diseases, University of Texas Health Science Center at San Antonio, San Antonio, TX USA; 9 Department of Biochemistry and Structural Biology, University of Texas Health Science Center at San Antonio, San Antonio, TX USA; 10 Groq, 400 Castro St #600, Mountain View, CA 94041 USA

**Keywords:** RHO signalling, Metabolic pathways

Correction to: *Nature Communications* 10.1038/s41467-021-26324-6, published online 19 October 2021.

This Article contained an error in Fig. 1. In Fig 1b, the GEVAL30 and GEVALNull labels for the images of both MDA-MB-231 and SK-Mel-103 cells were inadvertently switched.

