# Supplementary material for: Regulation of local GTP availability controls RAC1 activity and cell invasion
Source: Nat Commun. 2021 Oct 19;12:6091. doi: 10.1038/s41467-021-26324-6 (PMC8526568; doi:10.1038/s41467-021-26324-6)
Supplement: Supplementary file 1 — Supplementary Information [file 41467_2021_26324_MOESM1_ESM.pdf]

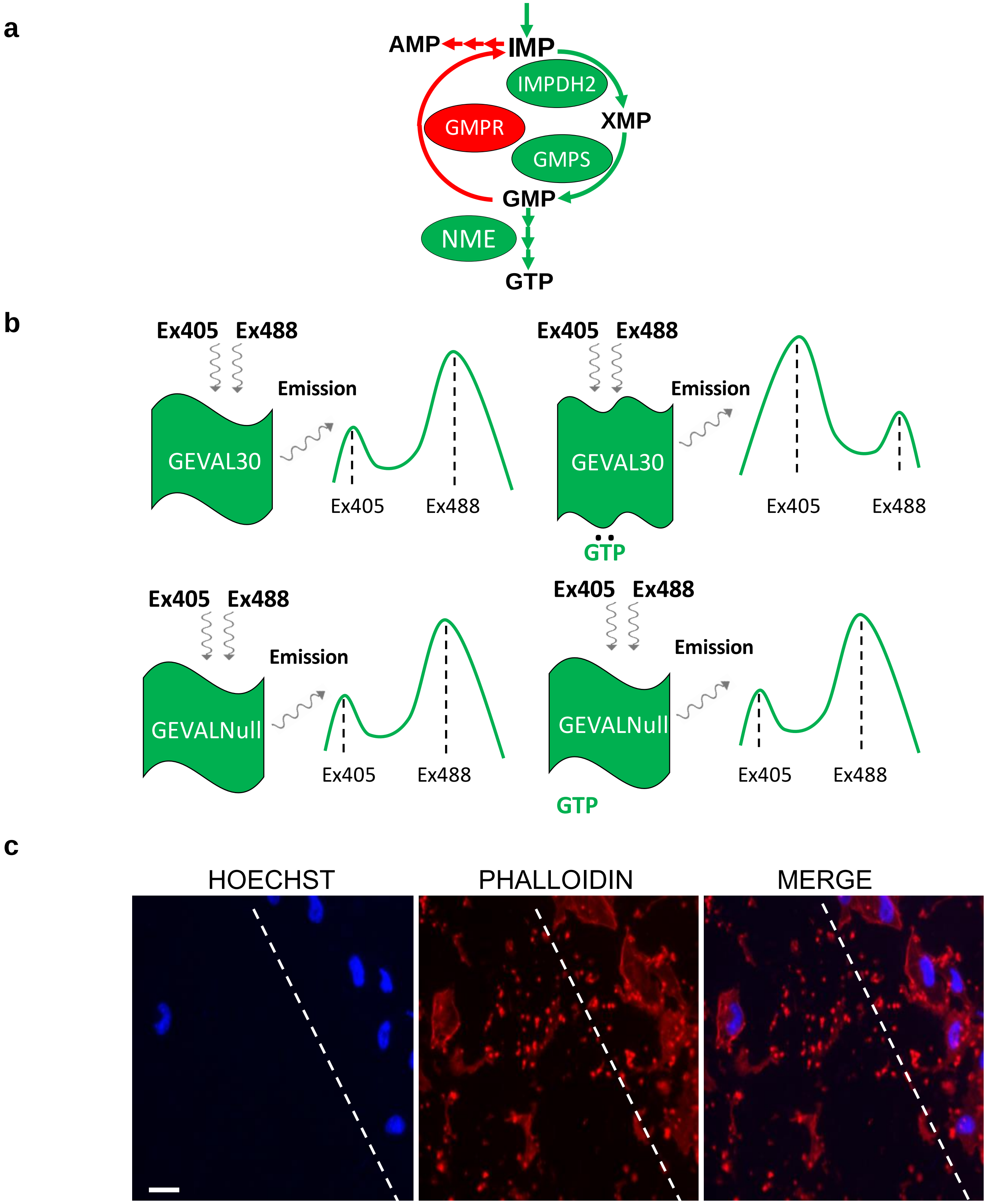

a. Schematic representation of the de novo GTP metabolism. **b.** Schematic presentation of ratiometric GEVAL sensors. GEVAL30 saturates with GTP at concentration of ~30μM. GTP binding to the GEVAL30 changes its conformation whereby decreasing fluorescence caused by excitation with 488nm and increasing fluorescence caused by excitation with 405 nm. The ratio of the emission signals at 488nm to 405nm is calculated. GEVALNull sensor does not bind GTP. **c.** Representative images of actin (red, phalloidin) and nuclei (blue, Hoechst) staining in MDA-MB-231 fixed in 4% PFA after cell protrusions induction. Part of the cell bodies compartment was swiped out with a cotton swab to verify the presence of actin puncta indicative of protrusions. Scale bar = 20μm. Representative images from one of 3 independent experiments.

Supplementary Fig. 1

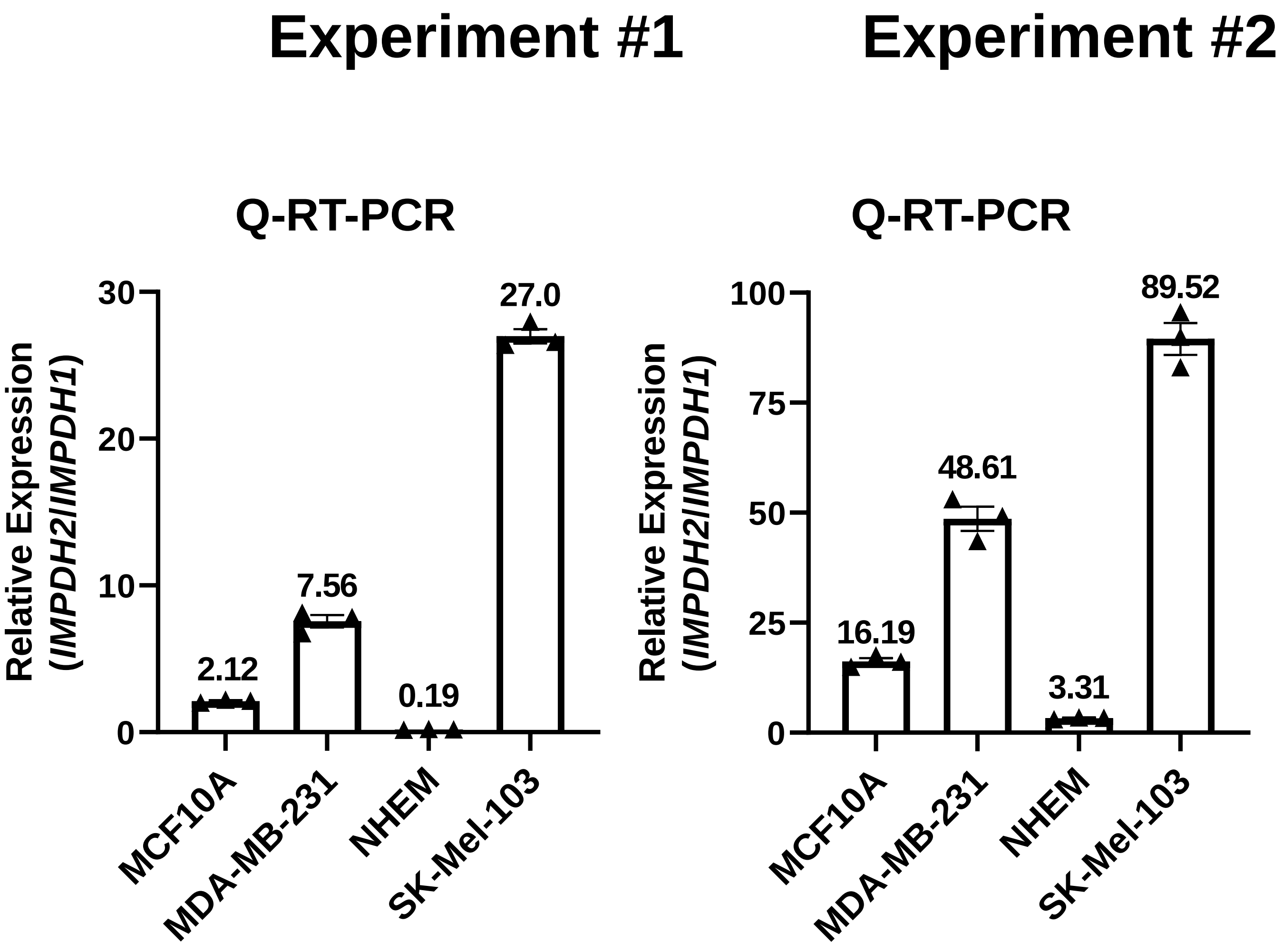

Indicated cells were probed in Q-RT-PCR with probes for *IMPDH1*, *IMPDH2* and *β-actin*. Shown are ratios of *IMPDH2* to *IMPDH1* levels after normalization to *β-actin* levels. Data represent average -/+ SEM of 3 technical replicates. Experiments were performed on RNA independently isolated from the indicated cell population (normal human epidermal melanocytes, NHEM) and cell lines.

Supplementary Fig. 2

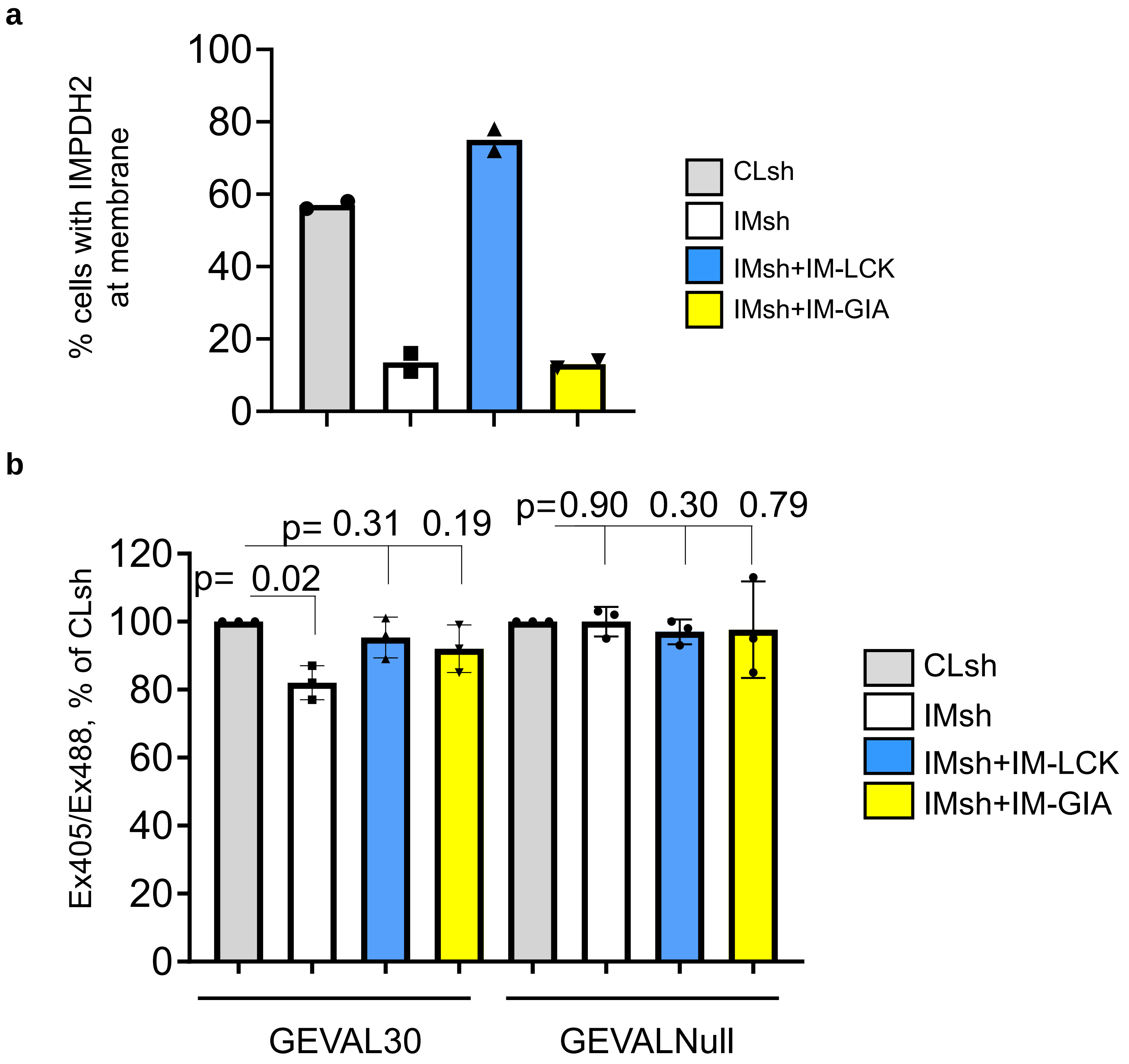

a. Quantification of the number of cells displaying presence of IMPDH2 at the cell plasma membrane, as assessed by immunofluorescence staining as is Fig. 2b. The data represents the average of 2 independent experiments. **b.** GEVAL-mediated analysis of intracellular GTP content in cells expressing the indicated constructs and super-transduced with either GEVAL30 or GEVALNull. The data represents the average -/+ Std Dev of 3 independent experiments. Statistics performed by two-tailed paired Student's t-Test.

Supplementary Fig. 3

SK-MEL-103

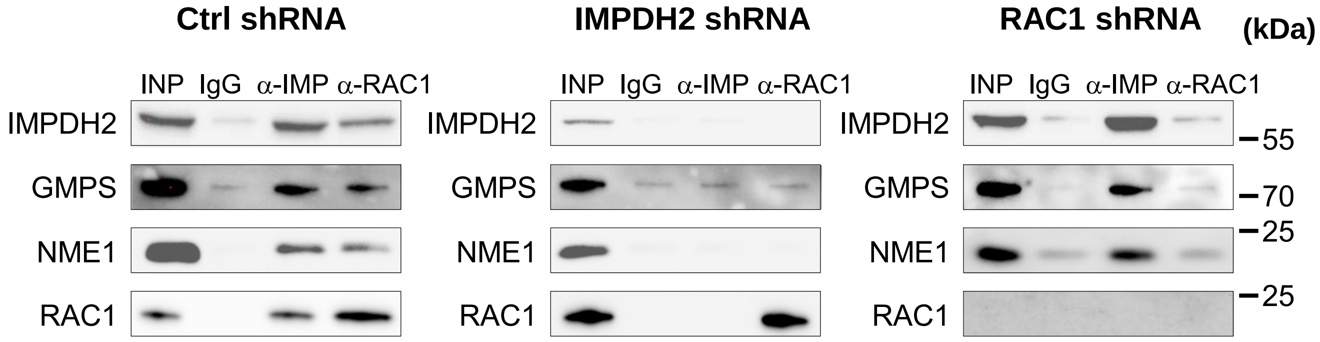

SK-Mel-103 cells were transduced with the indicated constructs, lysed and equal amounts of lysates were immunoprecipitated with the antibodies indicated on the top. The immunoprecipitated materials were probed in immunoblotting with the antibodies indicated on the left. Shown are representative images of at least two independent experiments.

Supplementary Fig. 4

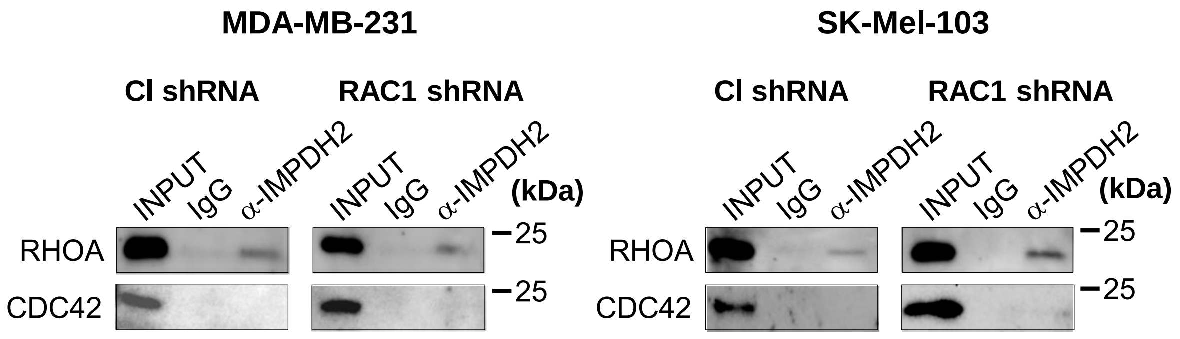

MDA-MB-231 and SK-Mel-103 cells were transduced with the indicated constructs, lysed and equal amounts of lysates were immunoprecipitated with the antibodies indicated on the top. The immunoprecipitated materials were probed in immunoblotting with the antibodies indicated on the left. Shown are representative images of two independent experiments.

Supplementary Fig. 5

a

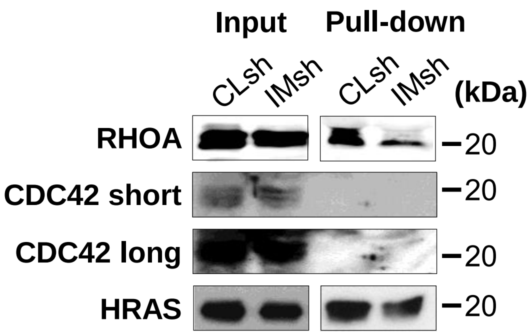

b

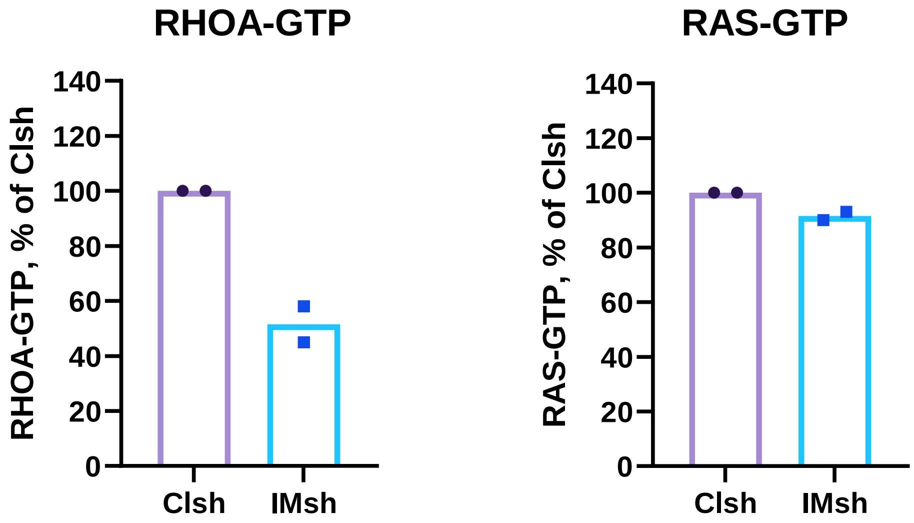

a. Cells transduced with the indicated constructs were probed in RHOA, CDC42 and RAS activity assay as described in Methods. Shown are representative images of two independent experiments. **b.** Quantification of (a). The data represents average of 2 independent experiments.

Supplementary Fig. 6

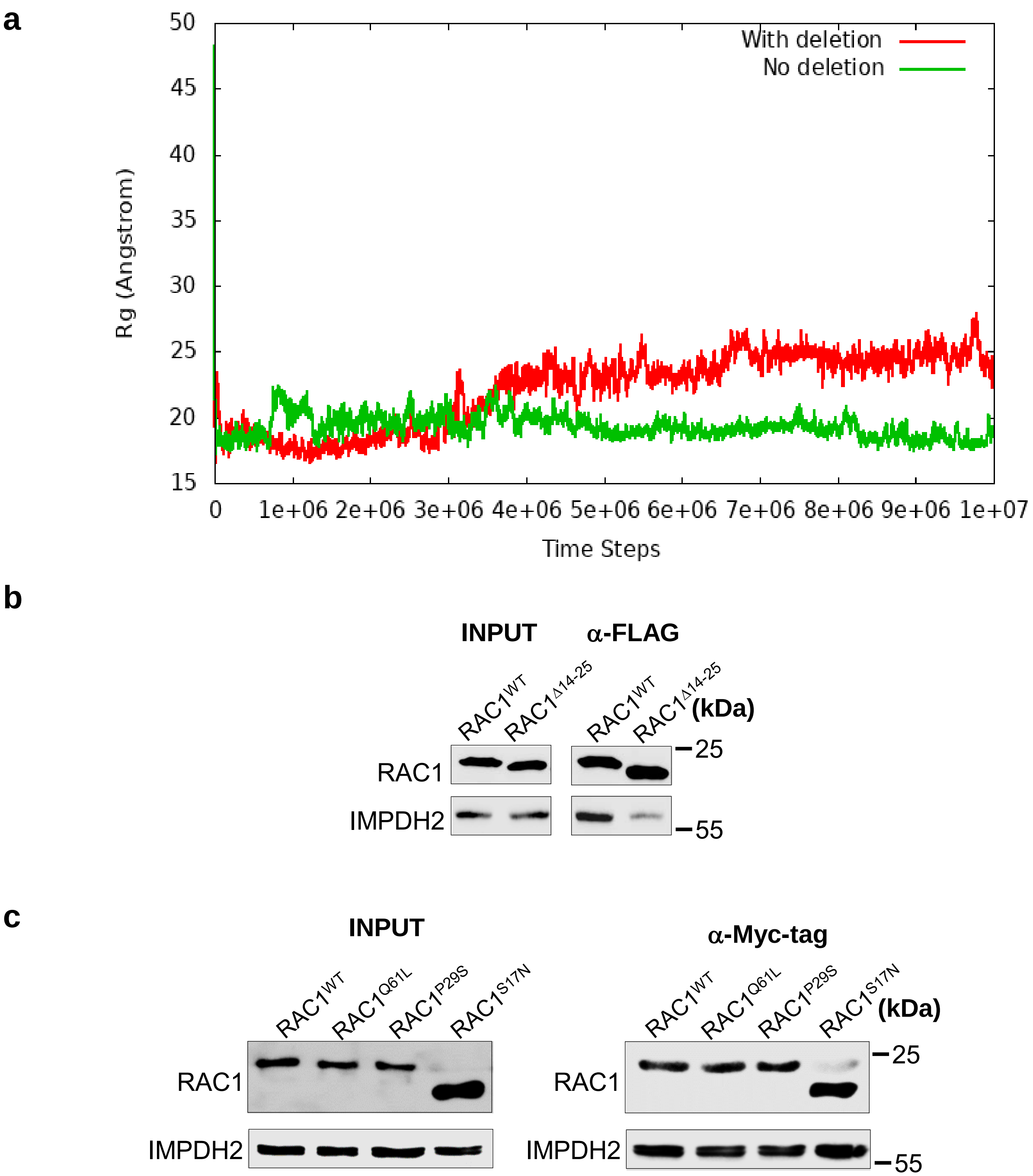

a. The radius of gyration variation as a function of the simulation time for the system formed by IMPDH2 and RAC1 (the wildtype is green and the mutant red). Twelve residues (amino acids 14 – 25) were deleted from the N-terminal of RAC1. **b.** MDA-MB-231 cells expressing the indicated constructs were subjected to in immunoprecipitation with the antibodies indicated on the top, followed by immunoblotting with the antibodies shown on the left. Shown are representative images of two independent experiments. **c.** Expression vectors containing indicated cDNAs fused to Myc-tag were transfected into HEK293 cells. Cells were subjected to immunoprecipitation with anti-Myc tag antibodies followed by immunoblotting with the antibodies indicated on the left. Please note that the difference in migration of $RAC1^{T17N}$ compared to other proteins is likely due to the fact that $RAC1^{T17N}$ is fused to one Myc-tag, whereas other RAC1 proteins – to two Myc tags. Shown are representative images of two independent experiments.

Supplementary Fig. 7

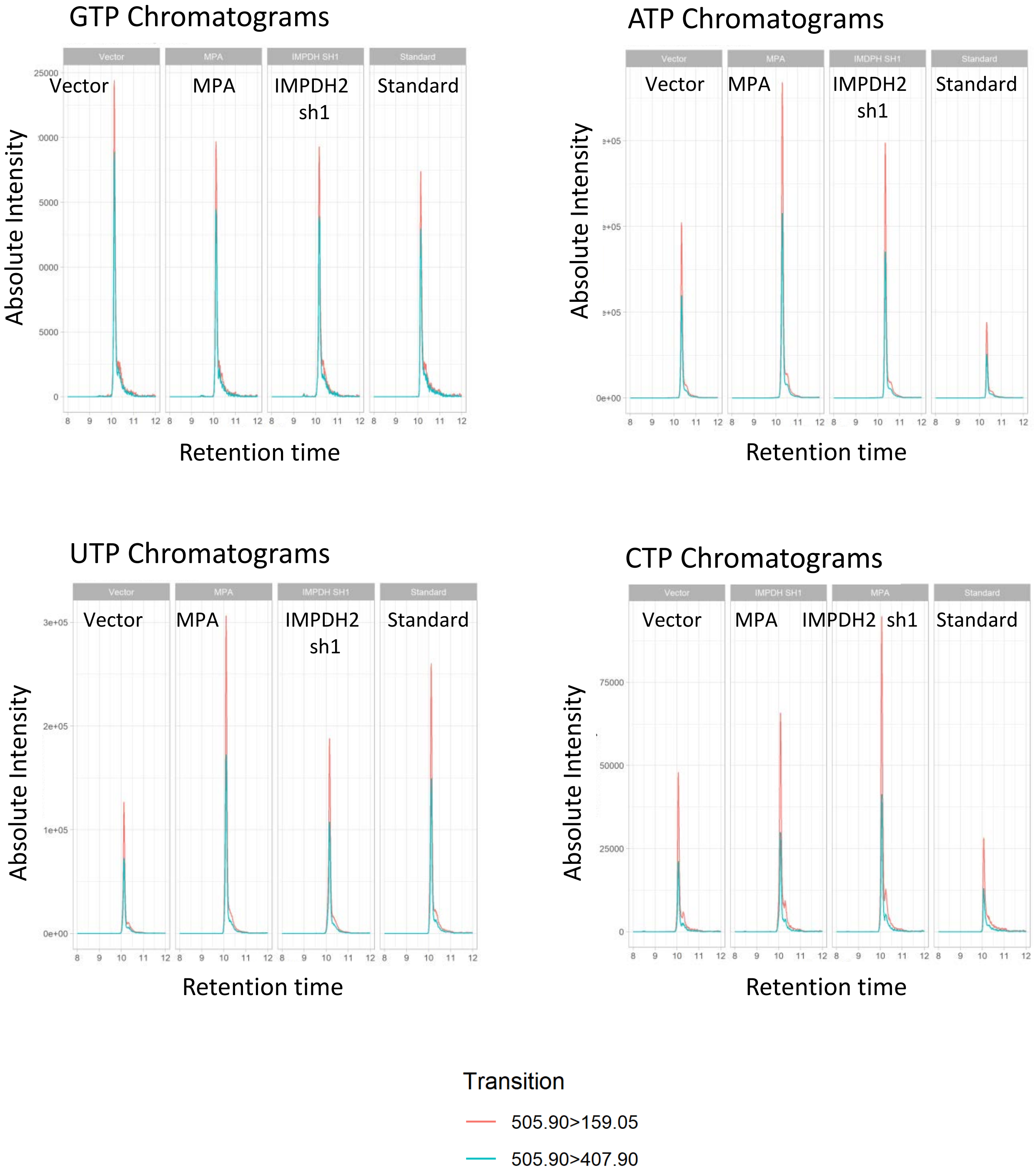

The chromatograms shown include two MRM transitions for each analyte species. The transition with the highest peak intensity was used for quantification and the second transition was used for retention time confirmation.
