# Supplementary material for: Regulation of local GTP availability controls RAC1 activity and cell invasion
Source: Nat Commun. 2021 Oct 19;12:6091. doi: 10.1038/s41467-021-26324-6 (PMC8526568; doi:10.1038/s41467-021-26324-6)
Supplement: Supplementary file 3 — Description of Additional Supplementary Files [file 41467_2021_26324_MOESM3_ESM.pdf]

Description of Additional Supplementary Files

File Name: Supplementary Movie 1

Description: The activity of GEVAL30 and RAC1 biosensors were measured with 1-minute intervals over the course of 30 minutes. Images in the video were rendered with preset “red hot” (for GEVAL index) and “magenta” (for FRET index) color maps in Fiji software78, using [0.45,0.85] and [0.25,0.70] scales, respectively.
