# Supplementary material for: Regulation of local GTP availability controls RAC1 activity and cell invasion
Source: Nat Commun. 2021 Oct 19;12:6091. doi: 10.1038/s41467-021-26324-6 (PMC8526568; doi:10.1038/s41467-021-26324-6)
Supplement: Supplementary file 5 — Reporting Summary [file 41467_2021_26324_MOESM5_ESM.pdf]

nature research

Corresponding author(s): Mikhail Nikiforov; Anna Bianchi-Smiraglia

Last updated by author(s): September 13 2021

Reporting Summary

Nature Research wishes to improve the reproducibility of the work that we publish. This form provides structure for consistency and transparency in reporting. For further information on Nature Research policies, see our Editorial Policies and the Editorial Policy Checklist.

Statistics

For all statistical analyses, confirm that the following items are present in the figure legend, table legend, main text, or Methods section.

n/a	Confirmed	
☐	☒	The exact sample size (n) for each experimental group/condition, given as a discrete number and unit of measurement
☐	☒	A statement on whether measurements were taken from distinct samples or whether the same sample was measured repeatedly
☐	☒	The statistical test(s) used AND whether they are one- or two-sided<br>*Only common tests should be described solely by name; describe more complex techniques in the Methods section.*
☒	☐	A description of all covariates tested
☒	☐	A description of any assumptions or corrections, such as tests of normality and adjustment for multiple comparisons
☐	☒	A full description of the statistical parameters including central tendency (e.g. means) or other basic estimates (e.g. regression coefficient) AND variation (e.g. standard deviation) or associated estimates of uncertainty (e.g. confidence intervals)
☐	☒	For null hypothesis testing, the test statistic (e.g. F, t, r) with confidence intervals, effect sizes, degrees of freedom and P value noted<br>*Give P values as exact values whenever suitable.*
☒	☐	For Bayesian analysis, information on the choice of priors and Markov chain Monte Carlo settings
☒	☐	For hierarchical and complex designs, identification of the appropriate level for tests and full reporting of outcomes
☐	☒	Estimates of effect sizes (e.g. Cohen's d, Pearson's r), indicating how they were calculated

Our web collection on statistics for biologists contains articles on many of the points above.

Software and code

Policy information about availability of computer code

Data collection	QuantStudio (6 Flex System, Thermo Fisher Scientific); MetaVue (v 7.8..12.0)
Data analysis	Image J (1.47v), Fiji, Microsoft Excel (Microsoft 365), GraphPad Prism (v8.0.1), MATLAB (Mathworks, version 2020b), Metamorph (Molecular Devices Version 0.2), Pymol (v 2.0), Graphpad (v 9.1.0), Proteome Discoverer (v 2.2), Coot-CCP4, SeqestHT and Xlinx v2.0 were both integrated in Proteome Discoverer 2.2.

For manuscripts utilizing custom algorithms or software that are central to the research but not yet described in published literature, software must be made available to editors and reviewers. We strongly encourage code deposition in a community repository (e.g. GitHub). See the Nature Research guidelines for submitting code & software for further information.

Data

Policy information about availability of data

All manuscripts must include a data availability statement. This statement should provide the following information, where applicable:

- Accession codes, unique identifiers, or web links for publicly available datasets
- A list of figures that have associated raw data
- A description of any restrictions on data availability

The experimental data generated in this study are provided in the Source Data File. Source data is provided with this paper.
The following databases/datasets have been used in this study: Swissprot Homo sapiens sequences RAC1 (P63000), IMPDH2 (P12268) PDB id: 6I0M, Kalirin DH1 domain PDB is: 5033. GENEBANK.
The protein mass spectrometry data has been deposited at XXX under accession code YYY.

Field-specific reporting

Please select the one below that is the best fit for your research. If you are not sure, read the appropriate sections before making your selection.

- [x] Life sciences
- [ ] Behavioural & social sciences
- [ ] Ecological, evolutionary & environmental sciences

For a reference copy of the document with all sections, see nature.com/documents/nr-reporting-summary-flat.pdf

Life sciences study design

All studies must disclose on these points even when the disclosure is negative.

Sample size	With a sample size of n=3 (to 9), we have 80% power (at α=0.05) to detect a difference of at least 3.1 (to 1.4) standard deviations (SD) between two groups
Data exclusions	No exclusions
Replication	Experiments were repeated multiple times (exact n described in each figure legend) and were reliably reporducible
Randomization	For in vitro studies, randomization is not applicable as cells with different treatments or genetic knockdown cannot be randomized.
Blinding	The investigators were blinded to group allocation during experiments and outcome assessment.

Reporting for specific materials, systems and methods

We require information from authors about some types of materials, experimental systems and methods used in many studies. Here, indicate whether each material, system or method listed is relevant to your study. If you are not sure if a list item applies to your research, read the appropriate section before selecting a response.

Materials & experimental systems

n/a	Involved in the study	
☐	☒	Antibodies
☐	☒	Eukaryotic cell lines
☒	☐	Palaeontology and archaeology
☒	☐	Animals and other organisms
☒	☐	Human research participants
☒	☐	Clinical data
☒	☐	Dual use research of concern

Methods

n/a	Involved in the study	
☒	☐	ChIP-seq
☒	☐	Flow cytometry
☒	☐	MRI-based neuroimaging

Antibodies

Antibodies used	IMPDH2 (Abcam, ab129165), RAC1 (Santa Cruz Biotechnology, sc-514583), Pierce™ Anti-c-Myc Magnetic Beads (ThermoFisher Scientific, 88842). Immunoblotting was performed with the following antibodies: RAC1 (Proteintech, 24072-1-AP 1:500), GMPS (Abcam, ab228716 1:1000), IMPDH2 (Proteintech, 67663-1 1:500), Tubulin (Proteintech, HRP-66031 1:1000), GMPR (Proteintech, 15683-1-AP 1:1000), NME1 (Proteintech, 11086-2-AP 1:1000), ITGB1 (Proteintech, 26918-1-AP 1:1000), KLF9 ((Santa Cruz biotechnology, sc-376422 1:250), RHOA (Cell Signaling, #2117, 1:500), CDC42 (Cell Signaling, #8747, 1:200), RAS (Cell Signaling, #8832, 1:500).
Validation	Per manufacturer: all antibodies in this study are validated by the manufacturers to be reactive to human proteins.<br>Previous cited papers and our own data antibodies validity has been tested in samples where the target proteins were depleted (i.e via shRNA) or over-expressed. Examples of validation reported in this paper; IMPDH2 (Fig. 2 and 4); RAC1 (Fig. 4).

Eukaryotic cell lines

Policy information about cell lines

Cell line source(s)	SK-Mel-103 human melanoma cells were obtained from Sloan Kettering Memorial Cancer Center. MDA-MB-231 human breast carcinoma cells were obtained from ATCC. HEK293T were purchased by Clonetech.
Authentication	MDA-MB-231 cells have been authenticated by STR genotyping at Roswell Park Comprehensive Cancer Center. SK-Mel-103 cells were authenticated at Sloan Kettering Memorial Cancer Center. HEK293T were authenticated at the vendor.
Mycoplasma contamination	Cells tested negative for mycoplasma contamination wit he the MycoAlert mycoplasma detection kit (Lonza)

Commonly misidentified lines
(See ICLAC register)

No commonly misidentified cell lines were used in this study
